# An Interventional Call-Back Service to Improve Appropriate Use of Antibiotics in Community Pharmacies

**DOI:** 10.3390/antibiotics10080986

**Published:** 2021-08-16

**Authors:** Bridget Paravattil, Monica Zolezzi, Ziad Nasr, Maria Benkhadra, May Alasmar, Sara Hussein, Aya Maklad

**Affiliations:** College of Pharmacy, QU Health, Qatar University, Doha P.O. Box 2713, Qatar; mzolezzi@qu.edu.qa (M.Z.); znasr@qu.edu.qa (Z.N.); mariabk1997@gmail.com (M.B.); mayelasmar.me@gmail.com (M.A.); sarayasserha@gmail.com (S.H.); am1402005@gmail.com (A.M.)

**Keywords:** community pharmacists, antimicrobial resistance, antibiotic adherence, antimicrobial stewardship, counseling

## Abstract

Pharmacists play a key role in tackling antibiotic misuse through counseling and education of patients and healthcare providers. The study aim is to evaluate the appropriateness of antibiotic prescriptions in community pharmacy settings while implementing an interventional call-back service to assess adherence and symptom resolution among patients prescribed an antibiotic. Patients were recruited by community pharmacists who were assigned to either the call-back, structured counseling, or standard care arms. Patients in the call-back group received intensive antibiotic counseling and a phone call from the study pharmacist 3 to 5 days after antibiotic initiation. The counseling arm patients received intensive antibiotic counseling from the study pharmacist while patients in the standard care arm received routine care. Antibiotic adherence rates among the standard care (*n* = 25), counseling (*n* = 29), and call-back (*n* = 26) groups were 64%, 86.2%, and 88.5%, respectively (X2 = 5.862, *p* = 0.053). Symptom severity scores after completion of antibiotic treatment among all groups were rated as excellent. Twenty-nine percent of the outpatient antibiotic prescriptions were deemed as inappropriate. A pharmacist call-back service is a simple and inexpensive intervention which can effectively identify opportunities for improving appropriate antibiotic use, particularly with respect to adherence.

## 1. Introduction

Antimicrobial resistance (AMR) is a major global concern that continues to rise at an alarming rate while posing as a significant threat to public health and safety [1,2]. Resistance arises from various mutations and changes in genetic material that renders pathogens less susceptible to several classes of antimicrobials. Consequently, infections become harder to treat and rates of spread, severity of illness, and mortality increase significantly [1,3].

Misuse of antibiotics is a known culprit of antibiotic resistance [4]. It is estimated 20% to 50% of antibiotics are unnecessarily or inappropriately used in the United States [5,6]. Likewise in Qatar, 45% of antibiotics for the treatment of upper respiratory tract infections were found to be prescribed for an inappropriate indication [7]. Inappropriate prescribing places patients at increased risk of Clostridioides difficile infections, adverse events, and allergic reactions which lead to excessive use of health care resources [8,9]. This type of antibiotic misuse promotes the development of multi-drug resistant organisms that are not only appearing in hospitals but are emerging in community settings [3]. 

Several regulatory bodies globally mandate the implementation of antimicrobial stewardship programs (ASPs) to improve the judicious use of antibiotics in hospital and community settings [9,10]. Although pharmacist-directed ASPs have proliferated considerably in the past decade, most of them are directed to improving antibiotic prescribing in hospitalized patients. Considering the impact of antibiotic resistance on the patient’s illness and the burden on society, community pharmacies are ideal for implementing ASPs [11]. Community pharmacists are essential antibiotic stewards and play an important role in providing information, education, and counseling to patients and health care providers on the appropriate use of antibiotics [12].

As community pharmacists embark on opportunities to enhance ASPs in the outpatient setting, studies have demonstrated the success of community pharmacists in improving antibiotic use among patients with interventions such as telephone follow-ups and written and oral communication [13,14,15]. Although these interventional approaches have been beneficial in North America, the appropriateness of prescribed outpatient antibiotics as well as the utility of an interventional call-back service has not been assessed outside this region. The practice of pharmacology within the community setting varies worldwide. Many pharmacies are not equipped with technology to access or retain the patient health records that allow for thorough review of patient clinical data and medication profiles. The majority of the medications are available for purchase without a prescription or consultation with a pharmacist. Additionally, community pharmacists continue to be heavily involved in medication-centered roles of dispensing with limited opportunity to perform patient-centered care services [16]. As such, examining the implementation of an antibiotic call-back service within community pharmacies that are comparable to those found in African, Middle Eastern, and Asian countries, is warranted. Therefore, the overarching aim of this study is to evaluate the appropriateness of antibiotic prescriptions in community pharmacy settings in Qatar and to evaluate the implementation of an antibiotic call-back service to assess antibiotic adherence and symptom resolution among patients.

## 2. Results

### 2.1. Study Population

A total of 80 patients completed the study. Patient demographics are shown in Table 1. Most patients enrolled in the study were of Asian descent and were prescribed antibiotics for dental and upper respiratory tract infections. Among the antibiotic prescriptions reviewed to evaluate for appropriateness, 70.9% of the prescriptions were reported as appropriate for the indication written on the prescription or based on the symptoms reported by the patients.

### 2.2. Antibiotic Adherence Rates and Symptom Severity Scores

Antibiotic adherence rates among the standard care (n = 25), structured counseling (n = 29), and call-back (n = 26) groups were 64%, 86.2%, and 88.5%, respectively (X2 = 5.862, *p* = 0.053). Twenty (25%) patients reported having antibiotics remaining after the treatment course. The reasons for antibiotic nonadherence included forgetfulness (35%), delayed start (20%), symptom improvement (15%), ineffective therapy (10%), not reported (10%), using when needed (5%), and occurrence of a side effect (5%). All patients who reported using antibiotics on an as-needed basis and discontinuing the antibiotic after symptom improvement were from the standard care group.

The symptom severity scores (1 = poor, 2 = average, 3 = excellent) before antibiotic initiation were significant for all groups with median scores of 2, 1, and 1 for the standard care, structured counseling, and call-back groups respectively, (X2 = 15.98, *p* ≤ 0.001), while the median severity score after antibiotic treatment was 3 among all groups (X2 = 0.609, n = 80, *p* = 0.737). 

### 2.3. Call-Back Service 

During the call-back service, study pharmacists provided 15% of patients with side effect management counseling, 15% with antibiotic adherence counseling, and 3.8% were referred to the physician due to worsening symptoms. Time spent on the phone ranged from 1 to 10 minutes with a mean of 3.3 (SD = 2.388) minutes.

The call-back service was perceived as useful by 84.6% of patients. The majority of patients (80.8%) reported their experience with the call-back service as excellent and 76.9% reported they would utilize the service again. Pharmacists’ responses regarding the call-back service are shown in Table 2. All pharmacists agreed the call-back service was beneficial and added value for patients on antibiotic therapy without significantly increasing their workload. 

### 2.4. Pharmacist Counseling 

When assessing the patient’s perspective on the pharmacist provided antibiotic counseling, all patients reported that the counseling experience was informative (Table 3). Median scores for instructions on how to use the antibiotic were reported as excellent among the three groups while the counseling related to antibiotic side effects was reported as average by patients within the standard care group. Patients within the standard care (69.6%), structured counseling (100%), and call-back (92.3%) groups reported the counseling provided by the pharmacist as helpful when taking the antibiotic (X2 = 10.71, *p* = 0.002).

## 3. Discussion

The call-back and structured counseling study arms reported higher antibiotic adherence rates when compared to the standard care group. Published studies using telephone follow-up services have reported antibiotic adherence rates of 78–94% [15]. The adherence rates found in this study fall within the range of previous studies. The standard care group had lower antibiotic adherence rates as the counseling provided to these patients was at the discretion of the pharmacist. Patients in the standard care group stopped their antibiotic early or used it on an as needed basis. This finding may suggest that patients have a continued lack of understanding of the role of antibiotics as well as highlight the inexperience of community pharmacists in recognizing their role in reducing antimicrobial resistance. Community pharmacists have been identified as leaders in outpatient ASPs and should be aware of their responsibility in reducing AMR [17]. Despite this designation as antibiotic stewards, literature continues to report gaps in community pharmacists’ knowledge of antimicrobial stewardship activities that can be implemented in outpatient setting [18].

Pharmacists are key to providing education to patients. Studies have shown the positive effects of educating patients on appropriate antibiotic use [19,20,21]. In this study, educating patients on the proper use and role of antibiotics by community pharmacists in the structured counseling and call-back groups reported better adherence rates and appropriate antibiotic use compared to patients in the standard care group.

In addition to the pharmacist’s role as an educator, community pharmacists are vital in influencing prescribing habits among providers. In this study, 29.1% of antibiotic prescriptions were deemed inappropriate which is similar to a study conducted by Fleming and colleagues that reported 30% of outpatient antibiotic prescriptions were unwarranted [22]. Community pharmacists in this study did not intervene upon these findings which may suggest their limited knowledge in assessing the appropriateness of antibiotics based on the prescribed indication or symptoms presented by patients. Conversely, monitoring prescribing behavior may be challenging due to the limited collaboration between pharmacists and prescribers [23]. Other factors such as the absence of a reimbursement model or access to patient health records have been identified in literature as barriers to participating in antimicrobial stewardship activities linked to prescribing practices [24]. 

Globally, many community pharmacists continue to dispense antibiotics without a prescription and potentially impact the judicious use of antibiotics [25,26,27,28,29,30]. Community pharmacists should act as gatekeepers, however due to pressure from patients, the need to generate revenue, or long wait times at physician offices, many are compelled to continue this practice while perpetuating the vicious cycle of antibiotic misuse [31]. To assist pharmacists in the reduction of AMR, this study found that educating patients on proper use of antibiotics either through comprehensive counseling or a call-back service improves adherence rates which has been consistent with other published data. As regulating the prescription status for antibiotics may not be strictly enforced in many countries, community pharmacists can utilize these inexpensive approaches especially in resource limited countries as an initial step towards combating the war against AMR. 

The new knowledge gained from this study is that community pharmacists require targeted and continuous education and training in antimicrobial stewardship. Their roles extend beyond patient counseling and should focus on judicious prescribing of antibiotics. As access to clinical data is limited in many community pharmacies worldwide, antibiotic assessment is mainly restricted to subjective evaluation of patient responses rather than microbiological cultures. Nevertheless, strategies such as encouraging prescribers to write the diagnosis on the prescriptions, implementing delayed prescribing, point of care testing, and developing collaborative practice agreements can be viable options to encourage appropriate antibiotic use within community settings [32,33]. AMR is a global concern that cannot be fixed with healthcare professionals working in silo. Community pharmacists and general practitioners need to work collaboratively to reduce antibiotic misuse in the outpatient setting. 

A few aspects should be considered when interpreting the study findings. Although the test of interest was appropriate, the small sample size hinders the testing for assumptions and small differences. Secondly, antibiotic adherence was measured by self-reported pill counts. Patients may perceive being blamed for not fulfilling their prescribed regimen and may report false data either purposefully or accidently [34]. Lastly as access to patient clinical data was not available when reviewing the appropriateness of the antibiotic prescriptions, the evaluation may be based on subjective rather than objective data. 

## 4. Materials and Methods

### 4.1. Study Design and Setting

A multicentered, interventional study was conducted among six community pharmacies in Qatar. Each community pharmacy was randomly assigned to one of three study groups (standard care, structured counseling, or call-back). Two pharmacists from each of the selected pharmacies were invited to participate in the study. Standardized instructions and training were provided to the pharmacists based on their group assignment.

### 4.2. Study Process

Patients presenting a prescription to the community pharmacist for an oral antibiotic and who were available to be reached by phone were eligible for enrollment by the study pharmacist. Patients who presented with topical antibiotic prescriptions and did not speak Arabic or English were excluded from the study.

Study pharmacists assigned to the structured counseling group received standardized training from the research team on how to conduct a thorough patient and medication history and how to provide a comprehensive antibiotic counseling session to patients. Role plays and sample patient cases were used to facilitate the 2 h pharmacist training session. Pharmacists were required to counsel each patient enrolled in the structured counseling study arm on the following: medication name, indication, dose, frequency, duration, side effects, precautions, administration, and how to handle missed doses. Pharmacists were also instructed to educate all patients to complete the whole antibiotic therapy course even if their symptoms started to improve. A counseling booklet of all available oral antibiotics found in Qatar was prepared and provided for each study pharmacist to use as a reference while counseling patients. The comprehensive counseling was provided to each patient prior to dispensing the antibiotic. 

Study pharmacists allocated to the call-back group also received the same standardized comprehensive antibiotic counseling training as the structured counseling group with an additional one-hour training session on how to conduct the call-back service. During this training, pharmacists were coached and provided with interventional approaches on how to manage antibiotic nonadherence, side effects, and ineffective therapy. In addition to providing comprehensive counseling prior to dispensing the antibiotic, study pharmacists were instructed to call patients 3 days after dispensing the antibiotic with a 5 day therapy duration or call patients 5 days after dispensing for an antibiotic with a 7–10 day therapy duration. During the interventional call, pharmacists were required to ask patients about antibiotic adherence, side effects, and symptom relief. Pharmacists were also instructed to counsel patients on side effect management, offer techniques to improve compliance, and to reemphasize the importance of completing the whole course of antibiotic therapy. If symptoms were not improving after initiation of the antibiotic therapy, pharmacists were instructed to refer patients back to their medical provider for further evaluation.

Study pharmacists in the standard care group were instructed to provide care as routine for patients who presented to the pharmacy with an antibiotic. Pharmacists in this study arm did not attend any of the training sessions provided for the structured counseling and call-back study groups. Counseling for patients enrolled in this study arm was at the discretion of the pharmacist.

### 4.3. Data Collection

All study pharmacists regardless of study group assignment collected patient demographic information, symptoms (to determine indication for antibiotic use when not specified on the prescription), and symptom severity at time of presentation to the pharmacy. Since the residents of Qatar are from various economic, educational, and ethnic backgrounds, a simple 3-point Likert scale (1 = poor, 2 = average, 3 = excellent) was utilized to assess the severity of their symptoms. All patients enrolled in the study were followed up by the research team within 1–2 days after completion of their antibiotic therapy to assess for antibiotic adherence by asking about the number of remaining tablets, symptom severity score, and the satisfaction with the counseling provided by the study pharmacist. In addition, patients and pharmacists in the call-back group were also asked about their satisfaction with the interventional call-back service. Patients who were unable to be reached after 2 days were considered lost to follow-up and excluded from data analysis.

### 4.4. Data Analysis

Given the exploratory nature of this study, a convenience sampling method was used to target 25 patients for each study arm. Six community pharmacies were pre-selected to obtain a representative and diverse sample of residents residing throughout Qatar.

The Chi-Square test was used to analyze the antibiotic adherence rates among the study groups while the Kruskal–Wallis test was used to compare the symptom severity score and pharmacist provided counseling among the three study groups. Descriptive statistics were utilized to analyze the demographics, interventions by the call-back group, and satisfaction among patients and pharmacists. 

The appropriateness of the prescribed antibiotic was evaluated by an expert with infectious disease experience. Prescriptions were deemed appropriate based on the Sanford Guide to Antimicrobial Therapy 2020 [35], antimicrobial prescribing policy of Hamad Medical Corporation, symptom presentation, and clinical judgement. 

### 4.5. Ethical Approval

This study was reviewed and approved by Qatar University Institutional Review Board [QU-IRB 928-E18]. All study participants provided written consent prior to study enrollment. 

## 5. Conclusions

An antibiotic call-back service was favorable among patients and pharmacists. Higher trends of antibiotic adherence were observed among the structured counseling and call-back study groups whereby highlighting the value of the pharmacist provided education. The call-back service may be used as a simple and inexpensive intervention to effectively identify opportunities for improving antibiotic use, particularly with respect to adherence within outpatient settings. Community pharmacists need to be involved in continuous education and training while working collaboratively with general practitioners to improve the judicious use of antibiotics.

## Figures and Tables

**Table 1:** Study Participant Demographics.

Patient Characteristics	N	%
**Nationality**		
African	2	2.5
American	1	1.2
Arab	14	17.5
Asian	37	46.3
Qatari	18	22.5
**Gender**		
Male	49	61.3
Female	26	32.5
**Age**		
Child (0–17)	17	21.3
Adult (≥18)	42	52.5
**Indication for Antibiotic**		
URTI ^1^	24	30
LRTI ^2^	2	2.5
Otitis media	6	7.5
Urinary tract infection	2	2.5
Dental	25	31.3
Skin infection	2	2.5
GI ^3^ infection	1	1.3
Eye infection	1	1.3
Unknown	17	21.3

^1^ URTI = upper respiratory tract infection; ^2^ LRTI = lower respiratory tract infection; ^3^ GI = gastrointestinal.

**Table 2:** Pharmacist Feedback with the Call-Back Service *n* = 4.

**Call-Back Service Assessment**	**Strongly Agree**	**Agree**	**Neutral**	**Disagree**	**Strongly Disagree**
The antibiotic call-back service added value for my patients.	2 (50%)	2 (50%)	0 (0%)	0 (0%)	0 (0%)
This service should be implemented for all your patients who are prescribed an antibiotic.	2 (50%)	1 (25%)	1 (25%)	0 (0%)	0 (0%)
The time required to provide this service to patients did not significantly increase my current workload.	2 (50%)	0 (0%)	2 (50%)	0 (0%)	0 (0%)
The antibiotic call-back service increased my job satisfaction.	1 (25%)	3 (75%)	0 (0%)	0 (0%)	0 (0%)
**Call-Back Service Assessment**	**Excellent**	**Good**	**Average**	**Fair**	**Poor**
How would you rate your overall experience with the antibiotic call-back service?	1 (25%)	3 (75%)	0 (0%)	0 (0%)	0 (0%)

**Table 3:** Patient Feedback on Counseling Received by Pharmacists within Study Groups.

Counseling Assessment	Standard Care	Structured Counseling	Call-Back	*p* Value
How well did the pharmacist instruct you about how to take your antibiotic?	3	3	3	0.000
How well did the pharmacist explain possible side effects?	2	3	3	0.001

## Data Availability

The data presented in this study are available on request from the corresponding author. The data are not publicly available due to ethical reasons.
